# Interaction between Filler and Polymeric Matrix in Nanocomposites: Magnetic Approach and Applications

**DOI:** 10.3390/polym13172998

**Published:** 2021-09-04

**Authors:** Moises Bustamante-Torres, David Romero-Fierro, Belén Arcentales-Vera, Samantha Pardo, Emilio Bucio

**Affiliations:** 1 Departamento de Biología, Escuela de Ciencias Biológicas e Ingeniería, Universidad de Investigación de Tecnología Experimental Yachay, Urcuquí 100650, Ecuador; 2 Departamento de Química de Radiaciones y Radioquímica, Instituto de Ciencias Nucleares, Universidad Nacional Autónoma de México, Ciudad de Mexico 04510, Mexico; david.romero@yachaytech.edu.ec; 3 Departamento de Química, Escuela de Ciencias Química e Ingeniería, Universidad de Investigación de Tecnología Experimental Yachay, Urcuquí 100650, Ecuador; maria.arcentales@yachaytech.edu.ec; 4 Facultad de Ciencias de la Vida, Universidad Politécnica Salesiana, Quito 170702, Ecuador; lpardo@est.ups.edu.ec

**Keywords:** polymer, nanocomposites, magnetic nanoparticles, composites, biomedical applications

## Abstract

In recent years, polymer nanocomposites produced by combining nanofillers and a polymeric matrix are emerging as interesting materials. Polymeric composites have a wide range of applications due to the outstanding and enhanced properties that are obtained thanks to the introduction of nanoparticles. Therefore, understanding the filler-matrix relationship is an important factor in the continued growth of this scientific area and the development of new materials with desired properties and specific applications. Due to their performance in response to a magnetic field magnetic nanocomposites represent an important class of functional nanocomposites. Due to their properties, magnetic nanocomposites have found numerous applications in biomedical applications such as drug delivery, theranostics, etc. This article aims to provide an overview of the filler-polymeric matrix relationship, with a special focus on magnetic nanocomposites and their potential applications in the biomedical field.

## 1. Introduction

Only a few years ago, the use of nanotechnology was considered to expensive for practical use. Nowadays the synthesis of nanoparticles (NPs) for different purposes in biotechnology and medicine has become a reality. The versatility of NPs has revolutionized a significant number of industrial and research areas. NPs are a class of materials with at least one dimension ranging from 1 to 100 nm. Magnetic nanoparticles (MNPs), represent one of the main classes of NPs which have attracted enormous interest owing to their biomedical applications such as biosensors, drug delivery, theranostics, imaging, and bioremediation. MNPs constitute an exciting class of nanomaterials that can be manipulated under the influence of an external magnetic field [1]. Scientists and materials scientists have shown tremendous interest in the properties of magnetic materials on the nanometer scale, while life scientists are also benefiting from the special properties of nanomagnets [2]. These properties can be further enhanced by taking advantage of the synergy with another biomolecule, such as a polymer.

Polymers are macromolecules with interesting properties depending on their nature and the polymerization process. These macromolecules serve as a matrix for the deposition of various materials at a nanoscale. For example, MNPs can interact as reinforcement in a polymeric matrix (PM), forming polymer nanocomposites (PNCs). The PNCs alters the microscopic dynamic processes due to the synergetic combination, leading to unique macroscopic properties.

The dynamics of polymers and MNPs are essential for understanding, developing, and manufacturing new composite materials. Thanks to the knowledge of polymer dynamics, the performance of these new PNC materials can be controlled. For example, it is known that mechanical properties, including stiffness, strength, and stress relaxation, are affected by segmental dynamics. Moreover, the assembling of magnetic nanoparticles on different polymeric matrices with controlled size, and properties has been extensively studied for the preparation of novel materials with outstanding properties [3]. This review provides a brief overview on nanocomposites and the interactions between fillers and polymeric matrices, with emphasis on magnetic nanocomposites and their applications.

## 2. Composites

A composite material is a multiphase material composed of two or more phases with different chemical properties [4], one of which serves as a matrix and the other as a reinforcing material. Combining the used reinforcement material and the matrix supporting the reinforcement material in the composite material gives it its performance and facilitates possible applications. Composite materials achieve the most beneficial properties from a strong bond between the robust and stiff reinforcement—usually fibers (filaments) or reinforcements with other geometrical shapes—for example, particles or platelets—and the weaker, less stiff matrix [4]. The wide variety of polymeric materials available, of both thermoplastic and thermosetting nature that can be tailor-made to meet specific performance characteristics, necessitates a clear identification of the property and processing requirements of the material to be used as a matrix in the composite [5]. Depending on the size, the composites can be defined as normal or nanoscale.

### 2.1. Polymer Nanocomposites

Studies related to nanotechnology have increased in the last years due to its versatility when applied in medicine. Nanocomposites have two parts: (1) a continuous phase and (2) a discontinuous or reinforcing phase [6]. PNCs consist of a polymer matrix as continous phase, with a nanoscale (more specifically less than 100 nm in size) reinforcing dispersed phase [7]. It is important to note that nanoparticles added to a continuous phase not only act as reinforcement, by NPs can modify one or more properties of the continuous phase [8]. According to their geometry and size these particles are defined as one-dimensional, linear, two-dimensional, layered, or three-dimensional, and powder NPs [9]. Similarly, nanofillers can be classified according to their chemical properties, physical structure, and particle shape. Depending on the nanomaterials used, other performance characteristics result. The inclusion of nanomaterials is directly related to the properties of the resulting polymer materials, changes the surface chemistry, and adjustments of the physical and chemical complexity of the polymer matrix [10]. The mechanical, thermal, electrical, and magnetic properties of polymeric nanocomposites are highly dependent on the interactions between the nanofillers and the PM. When the filler is dispersed in the polymer matrix, the surface area of the filler will form an “interaction zone,” leading to changes in polymer behavior, morphology, space charge distribution, and bond dispersion [11].

### 2.2. Principles

As previously mentioned, PNCs are materials produced by introducing NPs into a polymer matrix. After adding NPs to the PM, a nanocomposite is obtained, which will present a specific structure with physical properties that depend on the content and type of nanometric charge incorporated. The crucial aspect for developing a nanometric-size composite material is the adequate dispersion of the NPs to prevent their agglomeration in the matrix [12].

The analysis of the advantages and disadvantages of each of the existing processing methods allows determining which of them may be the most appropriate one for a particular application, considering the dispersion or distribution of particles, their processability and the physical-mechanical properties of the nanocomposites obtained.

The main components of the matrix can have a lipid origin (phospholipids, fatty acids), protein (collagen), natural polymers (chitosan, dextran), semisynthetic (cellulose derivatives) and synthetic poly ((acrylates), (acrylamides), (anhydrides) and (esters)) [13,14]. The chemical structure of the polymer will determine the behavior in terms of encapsulation, degradation, and release of molecules [13]; for example, amphiphilic polymers self-assemble in a structure with a covered hydrophilic core that serves as a repository for genes, enzymes, and a variety of drugs with different characteristics [15], combining the design with multiple functionalities while maintaining the nanoscopic scale [16].

Nanofillers have a high surface area. A larger interface area also means a more significant interaction area, therefore affecting the polymer’s behavior and morphology changes. It is important to note that the nature of the interaction between the filler and the matrix determines the magnitude of the effect produced by the presence of the filler. The interaction between the charge and the polymer is a complex phenomenon involving several interaction mechanisms that are unique to the complex according to its type, origin, size, shape, and preparation [11]. These interactions include electrostatic interactions, diffusion, surface tension, absorption, mechanical interlocking, chemical bonding, intermolecular bonding and surface wetting.

### 2.3. Effect of the Polymer

One polymer feature that profoundly affects segmental elements in PNCs is chain stiffness, probably because it emphatically influences interfacial pressing. In recreations of coarse-grained MD, the strength of the polymer chain is limited by the changing torsional potential (without changing other polymers or PNC limits), and the elements have been estimated almost like a level attractive substrate [17] that reproduces an interesting NP-polymer interaction. The segmented elements of the chain closer to the interface are slower than the mass and hardly care about stiffness. Nevertheless, the stiffer chains are affected by retreat, and the length scale is enlarged. Disconnecting the chain firmness is a test because changing polymer science will also change NP-polymer communication [18].

## 3. Role of NP-Polymer Interactions in Interfacial Dynamics

When a solid phase (NPs) is added to a polymer matrix, the segmental dynamics will be conditioned by certain factors that are related to each other, among which the perturbations in the polymeric conformations, the energy of the solid phase, and the difference of densities between the solid and the matrix can be highlighted [19]. Regarding the second point on energy, there is an enthalpy value related to the NP-polymer affinity and an entropic value referring to the conformation of the free polymer chains near the surface of the solid. Adjusting these values is possible through chemical modifications or material selection (enthalpy) and interfacial smoothness (entropy). However, this produces abrupt changes in other vital parameters, which complicates their analysis [20]. For example, it is known that a change in the interaction between the phases is affected, among other factors, by how the selection of material affects the stiffness of the chain, the quality of the solvent during preparation, and how the solid phase is dispersed [21].

Several experiments have been carried out to evaluate the role played by the NP-polymer interaction through modifications in each phase. In this way, composites with different states of aggregation and dispersion of the NPs have been obtained. This makes comparative analysis of interfaces complicated [22]. Thus, Schadler et al. determined that the work of adhesion and polymerization of the NPs could determine how they will disperse within the PM [23]. Thus, when the NP-NP interaction is less than the NP-polymer interaction and the relative work of adhesion is reduced, a fine solid dispersion is obtained. Thermal analysis has also found a higher T_g_ of the resulting composite [24]. This study represents a significant step at the moment to perform a priori nanocomposite property prediction. A method that allows access to the interfacial dynamics in repulsive interfaces while avoiding the aggregation of NPs in composites uses a polymer infiltrated in nanopores with well-characterized surfaces [25]. In the case of poly (ethylene-*co*-propylene) with a hydrophobic character infiltrated in the nanopores of aluminum oxide NPs with a hydrophilic character, the segmental dynamics was determined through neutron dispersion, and when compared with polydimethylsiloxane in AAO nanopores, both hydrophilic, the former shows more negligible segmental dynamics than in bulk [26]. In order to understand the phenomenon of the NP-polymer interface, in this section, several factors that influence the dynamic processes within nanocomposites are analyzed.

### 3.1. Nanoparticles Diffusion in Polymer Melts

The study of dynamics within complex systems such as polymers has significant importance for the development of advanced materials with biomedical applications. A specific interest is the movement of NPs and drugs in these materials, which will influence the mechanical, structural, and application properties of the material, properties of the colloidal system, and its targeting of specific sites during drug release processes [27]. The diffusion coefficient *D_SE_* of a spherical particle can be expressed by Equation (1), where it is related to a radius *R* in a medium having a viscosity η at a temperature *T* given by the Stokes-Einstein relationship:

(1) $$D_{SE}=\frac{k_{B}T}{6\pi \eta R}$$

which is fulfilled when the environment surrounding the spherical particles is homogeneous with respect to the spheres [28]. When the diffusion of NPs in solutions and polymer melts is analyzed, this relationship is not fulfilled, which has led to several theoretical and experimental investigations to understand the mobility of NPs in polymeric matrices.

These experimental studies have found substantial differences between the diffusion coefficients obtained and those calculated using the Stokes-Einstein relationship. These variations are largely due to the dependence between the dimensions of the diffusing particle and the polymer matrix in which diffusion occurs and the mobility time of the particle.

The deviations in the theoretical and experimental values are mainly because the diffusion of NPs in minor displacements is influenced by the structure and dynamics of the molten polymer [29]. Thus, these differences are attributed to the large difference between the macroscopic viscosity of the molten polymers and the local viscosity at the scale of individual monomers in the polymer chain. These conclusions are consistent with certain modern concepts proposed to establish a connection between material science and the intermolecular interactions and movements that occur in them [30].

The studies made it possible to delimit the scope of the Stokes-Einstein relationship in terms of the description of the diffusion of particles in polymers. It was found that the relationship is genuine when the diameter of the particle is larger than the dimensions of the matrices [27]. These results have been confirmed by studies that have analyzed the effect of the surface modification of the particles on their translational and rotational mobility and the diffusion of NPs. The studies considered NPs with dimensions greater than the radius of inertia of the polymer chains [31]. However, in studies of NPs with dimensions comparable to or smaller than the scales of the polymeric system, it has been proven that as the small particles adhere to the polymer, they strongly bind to the polymeric chains, producing essential changes in the properties of the polymer final composite compared to the introduction of particles of larger dimensions (d~10–50 nm) [32].

One of the methods for studying the molecular dynamics of this type of system was presented by Sebastiao et al. [33]. Their research focused on using conventional and fast-field cycling NMR relaxation techniques in polymeric nanocomposite systems for the development and optimization of drug delivery systems. Nevirapine was loaded into polymer-based matrices such as polycaprolactone, polylactide, polyvinyl alcohol, and maize starch. Concise spin-lattice relaxation periods were assigned to the amorphous regions, and the relaxation dispersion was similar to that of melt polymers, clearly showing the molecular dynamics of the studied matrices. The study concluded that the NMR relaxometry technique provides a broad spectrum of frequencies, which allows obtaining information on the mobility within the polymer chains and the interactions between the matrix and the drug. In all the systems studied, the power-law spin-lattice relaxation time dependence on the frequency could be used to adjust the experimental results, allowing to the differentiation in the modifications that occur in a particular polymeric base. On the other hand, the inclusion of clay NPs and nevirapine could cause a compensation effect.

### 3.2. Non-Diffuse NP Dynamics

Non-diffusive NP elements have been generally revealed in XPCS in different PNCs frameworks and trial conditions [34,35,36]. Methodical estimations of ∼100 nm measurement PS-joined SiO_2_ NP relaxations in unentangled PS as a function of Mw uncovered a temperature reliance to the extending (or packing) type, on the middle of the road dissipating capacity (ISF) and the scaling of n [37]. For instance, sub-dissemination is seen at high temperatures, super-dispersion is seen at lower temperatures, and the hybrid is observed at 1.25 T_g_, where Brownian dispersion is noticed.

In another XPCS investigation on the SiO_2_, NPs were added and scattered to the 11 nm measuring dispersed in PMMA with subatomic weight fluctuations, where the Brownian propagation can be seen in the untangled dissolution. However, super dispersion can be seen in the weight of subatomic traps, and the NP speed is sensibly free of Mw. The perception of non-diffusive NP elements is also considered in PNCs that include weakened and focused NP volume parts, connected and uncovered NPs, and under different estimation conditions. Strangely, Brownian dispersion was also found in XPCS with varying frameworks of PNC and requirements. The research on abnormal NP elements in XPCS is still confusing. Super diffusion, similar to speed, may occur under different circumstances when the test encounters active power without resistance (ballistic movement) or when the test is related to slopes or fields [38]. Future investigations should also mean understanding the source of this non-proliferation behavior and continuously developing new and accessible technologies to effectively test NP motion on a comparative time scale and directly compare it with XPCS.

## 4. Approaches to Nanotechnology and Polymer Research

The most significant advance in recent years in polymer chemistry research has not been the discovery of new monomers but rather the incorporation of nano-sized reinforcement additives. This technology gives rise to the so-called nanocomposites or hybrid organic-inorganic polymers. The fundamental characteristic of these hybrid polymers is that the inorganic additives are distributed in the polymer matrix on a nanometric scale, and, in the ideal situation, both phases are chemically linked [8,39,40,41]. If we compare nanocomposites with other traditional plastic filler materials, in the first case, the number of NPs that must be added to transform the properties of the material is significantly lower. This we should speak more appropriately of additives rather than filler materials.

By incorporating nanoadditives it is possible, for example, to simultaneously increase the toughness and resistance to breakage of plastic and improve its resistance to fire. It can also reduce the permeability to moisture and gases. This is important, for example, in barrier films for food use and adhesive joints, in which their performance in hot and humid environments is improved. For example, it has been shown that the addition of suitably functionalized NPs in an adhesive in amounts of the order of 5% allows doubling the resistance values to cutting and peeling of the adhesive. Despite the breadth of the advances obtained in nanocomposites, it is necessary to control the material’s size, stability, biocompatibility, and functionality, especially for biomedical applications related to detection and diagnosis, and therapeutic treatments. These factors must be considered to produce a new generation of materials that present maximum utility and minimal adverse effects when applied [42].

The potential market for nanocomposites is vast since it covers all those applications in which plastics and adhesive joints are used: containers and packaging, automotive, aerospace, biomedical, capital goods, etc. [43,44,45,46].

### 4.1. Dynamics in Polymer Nanocomposites

The study of dynamic properties plays a decisive role in the design, manufacture, and production of polymeric nanocomposites, influencing the transport of molecules (drugs, NPs, pharmaceutical agents, etc.) and the kinetic phase separation [47]. Polymer nanocomposites have improved material properties such as rigidity, mechanical strength, high-temperature resistance, weight reduction, corrosion resistance, and electrical conductivity. Moreover, their final properties depend on the kinetics of their constituent matrix enhancement, that is, the nature of the phase, the concentrated phase, the enhanced geometry (shape, size, and orientation), aspect ratio, dispersion state, and interface interaction.

This characteristic will depend on the material used in the filler. For example, research mentions that if the carbon nanotubes are higher than the “percolation threshold” named after the umbra [48], the carbon nanotubes in the polymer matrix will conduct electricity. If the materials used are different, the density and umbra value will change. When a material is transformed into a nanomaterial, the temperature is a physical property that will affect the material’s physical properties. Studies have shown that since the new composition of nanomaterials is much more than internal atoms, the melting point, transition temperature, and constant changes of the material on the nanoscale are possible [9].

### 4.2. Polymeric Matrix (PM)

A PM is a particular arrangement of polymers corresponding to the discontinuous phase. Almost all polymers, including thermoplastics, thermosets, elastomers, and biopolymers (including various biodegradable molecules), have been used to prepare PMs [44]. The matrix parameters include the type of polymer, the nature of the surface, the polymer’s chemistry, the volume or weight fraction, and the structure. One of the most common types of hydrophilic PMs are those known as hydrogels. A hydrogel is a tridimensional polymeric structure with swelling and collapse properties, flexibility, biodegradability, biocompatibility and softness [49]. The hydrogels are well known for their capacity to absorb drugs [50] as well as NPs.

Either hydrophilic or hydrophobic polymers can be used as a matrix to interact with another molecule. Co-casting of a hydrophobic polymer and a hydrophilic polymer is another method to make composite membranes with hydrophilic surfaces and high mechanical strength [51]. In general, the PM can be filled by a great wide of particles with a small size to fill the voids. For example, adding a small amount of NP reinforcing agent can substantially increase the strength and stiffness of the polymer and qualitatively improve its properties [52].

### 4.3. Filler

Filler parameters include filler size and type, superficial filling, filler volume or weight fraction and treatment. The reinforcing material used to prepare PM can be a crystalline structure, such as iron and other metal powders, clay, silica, TiO_2_, other metal oxides, or carbon-based nanomaterials, including fullerenes, carbon nanotubes (CNT), and carbon nanofibers [53] and NPs. Numerous combinations of the unit components of nanocomposites exist, which involve MNPs as fillers in a PM [54]. Typically, the filler is combined with the matrix material, and the volume fraction in the composite material is usually less than 50%. Filling or reinforcing materials with high strength and rigidity can provide the main characteristics of advanced composite materials and can be widely used in engineering. The loading of NPs can change the segmental dynamics and mechanical properties of the polymer matrix. For example, the addition of MNPs provides conductivity and magnetism in addition to embedded properties like reinforcement inclusion [55]. The magnetic properties of NPs are well used in the development of polymer matrix composites. In these materials, the NPs act as the reinforcing phase and are dispersed on the surface of the polymer matrix [56].

### 4.4. Effect of Filler

A typical feature to analyze in PNCs is the general measure of polymer and filler, which constitute the NP-polymer interfacial area [18,57]. Also, in specific PNCs frameworks, particularly at sufficiently high NP loads, NP-NP conglomeration and polymer crossing over contiguous NPs have non-insignificant consequences for segmental elements [58]. The instance of NP collection can regularly be considered as far as the NP-polymer interfacial region because aggregated NPs have extensively less open surface region to the grid (per unit volume NP) than separately scattered NPs. Accordingly, segmental elements are commonly less affected in accumulated PNCs than those with a similar volume portion of independently scattered NPs [59].

One fundamental feature of the filler that influences segmental elements is the NP size. Note that diminishing the NP size at a fixed NP load and arbitrary scattering state increases the NP-polymer interfacial region and diminishes the interparticle separation distance. Since segmental elements are frequently annoyed at the NP-polymer interface, suggesting that a more modest NP load will influence more portions in the PNCs [60].

As to the state of the NP filler, testing data are scant for the particularly eminent case of carbon-based NPs and clays. Entanglements result in restricted accessibility of non-circular NPs and changes in NP-polymer communication with changes fit as a fiddle. Be that as it may, we foresee NP shape to affect segmental elements principally through changes in the neighborhood interfacial compression, nearby sweep of ebb and flow, and measure of the polymer-open interface. Segregating and deconvoluting these impacts remains tentatively testing [21]. The use of magnetic modified polymers has increased in the last years due to the inherent properties of magnetic particles such as magnetic fields and low costs in manufacture. Usually, the magnetic particles are embedded in a polymeric network since it allows the rotation of the hybrid material as a stir-bar in the sample, favoring the kinetics of the adsorption and desorption of the analytes during the extraction [61,62,63]. The coating of the magnetic cores is crucial for protection against oxidation and particle aggregation [64].

## 5. Magnetic Nanoparticles (MNPs)

MNPs show excellent features such as small size, high surface area, the active surface that can successfully be modified, low toxicity, and superparamagnetism [65]. MNPs have received remarkable attention in the development and fabrication of biosensors and sensors for numerous applications, including the environment, foods, the clinic and biology [66].

Magnetic nanoparticles present unique physical properties at the cellular and molecular levels of the biological interface [67]. Indeed, magnetic nanomaterials possess the ability to penetrate through human tissue the magnetic field and manipulate it in such a way that they can be exploited for medicinal purposes [68]. However, they need to be functionalized by surface ligands such as polymeric coatings, metallic coats such as silica or gold, liposomes, and micelles, since individually, they are toxic and tend to be easily recognized and cleared out [66] by the organism.

MNPs can be obtained in several forms, compositions and phases, including iron oxides, pure metals (e.g., iron, nickel, chromium, cobalt, manganese), spinel ferrites, alloys, dilute magnetic semiconductors and polymer magnets [69]. The production of magnetic nanoparticles can be achieved through remarkable approaches such as co-precipitation [70], polyol [71], hydrothermal [72], thermal decomposition [73], microemulsion [74], sol-gel method [75], etc. The physical and chemical characteristics of magnetite nanoparticles can be controlled by varying the reaction parameters. Therefore, depending on the desired applications, magnetite nanoparticles with specific properties will be obtained. A number of studies on these processes for synthesis of magnetic nanoparticles have been reported in the literature [76,77]. The preparation of nanomaterials with controlled morphology, size, and dimensions is an essential target of advanced materials, especially to fabricate anisotropic inorganic nanomaterials with at least one dimensional material [78].

In comparison to spherical MNPs, magnetic nanowires (MNWs) possess more degrees of freedom in achieving magnetic, surface chemical tenability; besides the adjustment of magnetic anisotropy and inter-layer interactions, another critical feature of MNWs is their ability to combine different components along their length, which can result in diverse bio-magnetism applications [79]. The magnetic properties of the nanowires can be tuned by changing the shell thickness to yield remarkable new properties and multi-functionality [80]. MNWs have contributed significantly to the nanomedicine field because of their low toxicity and ease of manipulation with the magnetic field [81]. Furthermore, MNWs have shown a high internalization by cells compared to other magnetic nanoparticles, such as iron oxide nanoparticles, improving the enrichment and multiplex-ing yield [82,83,84]. Another important form of nanoparticles is the one known as nanosheets, which are arranged as a single- or multiple-layer two-dimensional array of atoms or molecules [85]. Nevertheless, the magnetic behaviors of the 2D nanosheets in isolated single-layer forms are unclear at the moment [86]. Saber and his collaborators used solvent and a thermal technique to prepare nanosheets with an α-Fe_2_O_3_ hematite structure, obtaining a particle size of 100 nm, with an excellent magnetite structure, which possesses superparamagnetic behavior and exhibits relatively high saturation magnetization [87].

### 5.1. Properties of MNPs

**Figure 1:**
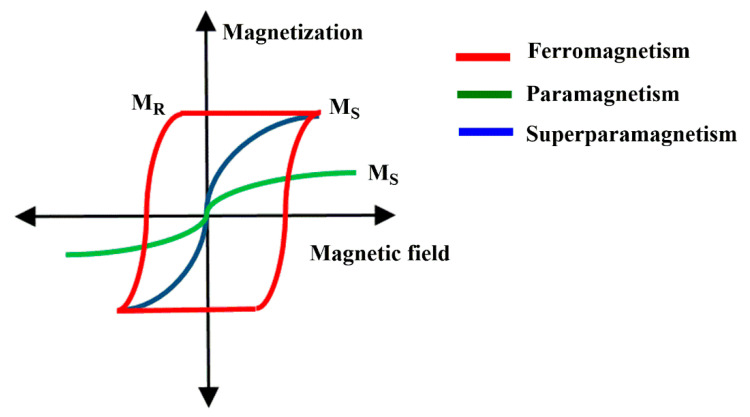
Comparison between magnetization curves of different types of materials: ferro-, para- and superparamagnetics.

**Figure 2:**
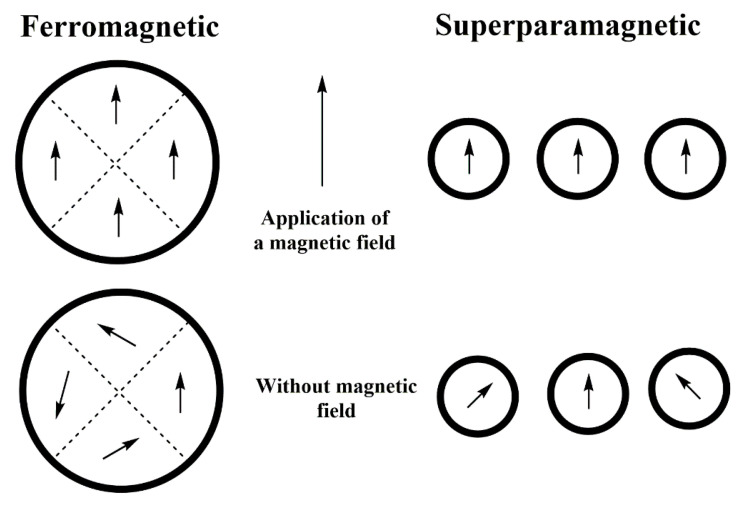
Effect of exposure to a magnetic field of ferromagnetic and superparamagnetic particles.

MNPs exhibits different behavior based on their response in the presence and absence of an applied magnetic field as shown in Figure 1. These properties are briefly explained below:

Ferromagnetism materials display an aligned atomic magnetic moment of equal magnitude. In these materials, even in the absence of any external magnetic field, the moments tend to be aligned parallel to each other, as shown in Figure 2. Hard magnets are materials that in the absence of an applied field exhibit permanent magnetization [88]. Concerning electronic configuration, ferromagnetism usually appears in materials with partially filled outer valence shells. Some examples of ferromagnetic materials are Fe, Ni, Co, and Cu [89,90]. When these substances are within a specific temperature range, there is a net atomic moment, and the arrangement is such that the magnetization continues after the external electric field is removed. Below a certain temperature called the Curie temperature (CT), the continuous increase of the magnetic field applied to the ferromagnetic substance will cause the magnetization to increase to a high value, called the saturation magnetization [91]. However, when the ferromagnetic body is heated, the parallel temperature will disappear when the temperature is above the CT leading to a paramagnetic behavior [92].

On another hand, ferrimagnetic materials contain magnetic moments oriented antiparallel to each other, which are not cancelled, resulting in a net spontaneous magnetization. Materials such as Fe_3_O_4_ and Fe_3_S_4_ are examples of ferromagnetic materials [90].

The superparamagnetic behavior is a magnetic state that can occur in ferromagnetic and ferrimagnetic materials depending on parameters such as size, magnetic anisotropy, and temperature [93]. Under the influence of the temperature, the magnetization may change direction randomly in small nanoparticles [94]. The number of magnetic domains in a particle generally depends on its size. When the particle has a radius less than a certain critical value (nanometric scale), the high value of the energy associated with the domain walls inside it is thermodynamically unfavorable, and the material becomes a monodomain in which all its magnetic moments are oriented in the same direction [95]. On the other hand, in the absence of an external magnetic field, the magnetic moment has two possible orientations in the same direction called the easy axis of anisotropy. Superparamagnetism plays a key role in biological applications such as drug delivery [96], where nanoparticles do not exhibit magnetic properties upon removal of the external field, and then do not attract each other, which eliminates the main force for aggregation.

Similar to paramagnetism, superparamagnetism occurs when the temperature is above a critical value, which in this case is known as the blocking temperature (BT) [95,97]. BT is defined as the limit temperature at which the thermodynamic equilibrium state and the blocking state separate [98]. While in paramagnetism, the CT is an intrinsic characteristic of the material, BT depends on several parameters such as the size of the NP and the shape of the NP that produces the so-called shape anisotropy. Above the CT, superparamagnetic materials exhibit similar behaviors to paramagnetic materials but with a much higher magnetic moment [98,99]. This can be observed in the magnetization curves of both materials, in which it can be seen that superparamagnetic materials have a much higher magnetic susceptibility than paramagnetic ones. Likewise, below the BT, the magnetization curve exhibits a hysteresis similar to the magnetization curve of ferromagnetic materials [94].

A ferromagnetic bulk material has a cooperative magnetic behavior, to decrease the magnetostatic energy, which is the potential energy produced by the external magnetic field. This behavior leads the atomic spins to line up in parallel in “sections” called magnetic domains, separated by regions called Bloch walls [95]. Within each domain, the spins are aligned in parallel, but the direction of the spins is different between domains so that they all compensate, and the result of the entire mass of material is zero magnetization. When the size is reduced (NPM for magnetite is less than 10 nm), the domain walls disappear from the material, and the atomic magnetic moments align in a single domain [97]. This change is fundamental since superparamagnetic behavior originates in NPs, which is defined by having a high magnetic susceptibility [95], but with the characteristic of being of turning the spins randomly under the influence of temperature. Moreover, magnetic nanoparticles also exhibit other interesting structural, optical, electrical, and gas sensing properties, which have been extensively reported in the literature [100,101,102].

### 5.2. Magnetic Nanocomposites

MNPs are attractive as reinforcing fillers, for which it is essential to control the size and shape of the NPs to ensure these are well dispersed within the PM [7,103]. Data analysis is much easier in magnetic nanocomposites containing particles of uniform size [104]. Nanosized particles of ferromagnetic and ferromagnetic materials have been incorporated into extended matrix materials to create integrated functional systems with additional magnetic properties [6]. Magnetic nanocomposites exhibit superparamagnetic behavior, which is a property highly recommended for biomedicine, through the use of an external magnetic field and thus the treating of complex diseases [10]. Magnetic nanocomposites (MNCs) exhibit superparamagnetic behavior, which is a property that can be used for the controlled and targeted drug delivery, through the use of an external magnetic field and thus treat difficult diseases [72]. Moreover, magnetic nanomaterials combining magnetic separation and nanotechnology would help remove heavy metal ions (bioremediation) [105].

Prabha and Raj developed a system to accomplish this in cancer treatment. They managed to load an anticancer drug in a polymeric nanocomposite of chitosan, polyethylene glycol, and polyvinylpyrrolidone with a superparamagnetic iron oxide nanoparticles (IONPs) coating. The charged particles in the different systems show an average particle size of between 183–390 nm and zeta potential of 26–41 Mv [104]. The combination of ferromagnetic and superparamagnetic-like behaviors of these core-shell NPs made them attractive for applications in nanomedicine, where they can be used to target or destroy cancer cells [10]. Also, thanks to their relatively large surface area and therefore high active surface sites, superparamagnetic NPs can absorb metal ions so that they can be very quickly removed from a matrix using a magnetic field and reused without losing the active sites [106]. As we mentioned before, hydrogel serves as a host media to interact with NPs. It allows for a high content in MNPs and avoids phase separation during exposure to a magnetic field [107]. Gao et al. developed a simple, nontoxic, water-based strategy to fabricate MNP/hydrogels nanocomposites in which highly crystalline Fe_3_O_4_ nanoctahedra can be fabricated in situ within a negatively charged hydrogel matrix [108]. Nicholar and his colleagues developed MNCs with MNPs covered by polymers. This method was developed through thermal decomposition of pentacarbonyl in the presence of ammonia and polymers polyisobutylene, polyethylene, or polystyrene chains functionalized with tetraethylenepentamine, a short polyethyleneimine chain [109]. Moreover, Wang et al. fabricated via the in-situ growth of nano Fe_3_O_4_ on the polydopamine (PDA)-functionalized MoS_2_ nanosheets, and subsequently applied as the solar absorber in the volumetric evaporation systems to generate solar steam, where the magnetic MoS_2_ nanosheets not only showed the well long-term dispersion in aqueous solution due to the introduction of hydrophilic PDA but also exhibited a fast and effective separation from aqueous solution with the help of the decorating nano Fe_3_O_4_, which much benefited to the continuously efficient solar steam generation and its good recyclability, respectively [110].

The MNPs are combined with different polymers and correspondingly different functional groups in their surfaces. Magnetic gels present a stimuli-responsive approach when are subjected to a magnetic field [111]. For example, Barbucci et al. developed smart hydrogels containing functionalized CoFe_2_O_4_ MNPs, covalently bound to a carboxymethylcellulose (CMC) polymer. The NPs serve as cross-linkers by linking CMC’s carboxylic groups and the amine groups of functionalized NPs [112].

## 6. Applications

### 6.1. Biosensors

**Figure 3:**
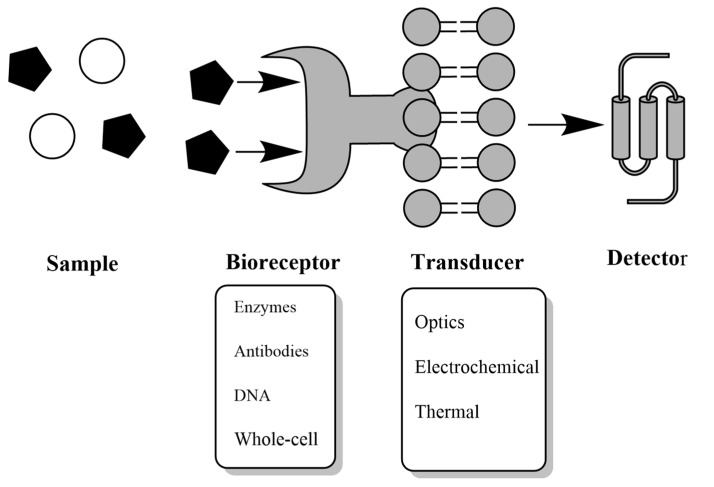
Scheme of a biosensor and rationale for its operation.

In recent years the importance of using biosensors as analytical methods in different fields has increased compared to the use of conventional alternatives. A biosensor is an analytical instrument that is made up of a biological receptor such as an antibody, enzyme, nucleic acid, and whole-cell; prepared to precisely detect a substance using the specificity of biomolecular interactions, a transducer or sensor, capable of interpreting the biological recognition reaction produced by the receptor and “translate” it into a quantifiable signal, optical or electrochemical, and a signal display or reading indicating the presence and concentration of or analyte molecules [113,114] (Figure 3).

The most striking features of these devices are that they make them an attractive choice for analysis tools: their specificity, high sensitivity, short response time, short analysis time, their ability to be included in an integrated system, and easy automation, strong real-time workability, versatility and low cost [115]. However, a problem in biosensor technology is the immobilization of the substance that makes up the biological receptor. For this, studies have investigated the use of conductive polymers as enzyme immobilizers. The incorporation of polymers allows controlling the spatial distribution of immobilized enzymes and modulating their activity by changing the state of the polymer, providing good detectability and a rapid response [116].

On the other hand, recent studies have investigated the use of MNPs in electrochemical biosensors, where the selectivity of these devices depends on the identification element and the host matrix. New approaches have analyzed the immobilization of glucose oxidase in reduced graphene oxide (RGO) covalently conjugated with MNPs (Fe_3_O_4_NPs). The results show a biosensor with high stability, good reproducibility, excellent selectivity, and a successfully applied detection potential at −0.45 V. This method of detecting glucose levels took advantage of covalent binding and self-assembly as a new approach to immobilize enzymes without the need for a binder. It is important to emphasize that this is a new method to manufacture excellent quality electrochemical biosensors [117]. Such is the case that magnetics polymer nanocomposites help achieve sufficient stability and sensitivity because their NPs act as redox mediators for biomolecules, while the polymers act as selective adsorbates for biomolecules [118].

Concerning environmental applications, nanocomposite biosensors can be used to discover specific types of harmful extensions of materials that exist or dominate in the environment, thus determining various pollutants, toxic intermediates, and heavy metals in waste or water sources [119]. For the detection of heavy metals and pesticides, the use of modified electrodes and conducting polymer is necessary; this conjugation allows the immobilized enzyme and quantifies the problem compound [120]. Furthermore, NPs in biosensors can also be used to determine water quality parameters, such as biological oxygen demand, the detection of nitrates and phosphates, contributing to the data necessary for bioremediation processes. For example, tyrosinase (Tyr) biosensors based on Fe_3_O_4_ MNPs are used to detect the concentration of fecal coliforms, also contributing to the analysis of water quality to clinical diagnoses. However, it should be studied under different concentration conditions, environment, and detection limits to obtain better results [121].

### 6.2. Drug Delivery

The increase in diseases in recent decades, as well as their complexity added to the toxicity of certain drugs to treat them, has led to the search for new technologies that optimize drug administration systems, which should aim to use decreasing doses and increasing efficiencies, with the fewest possible side effects [122,123]. This can detect pathological conditions more effectively at an early stage, thus improving the patient’s condition. However, improved drug delivery systems (DDS) must meet the following criteria: a simple route of administration, effective targeting and delivery of the drug to the site of administration, and they must be biospecific, non-toxic, biocompatible, and biodegradable [123].

Polymers have attracted attention due to their properties such as biocompatibility, straightforward design and preparation, variety of structures, and interesting biomimetic character [124], and those added with reinforcements on a nanometric scale have aroused curiosity within the area of drug administration due to the specific delivery to the site of action that they provide, in addition to the versatility they have to meet the requirements of each administration system in particular [123,124].

In the field of intelligent drug administration, the use of polymeric nanocomposites is crucial because NP provide properties such as offering a greater carrying capacity, biodegradability, traveling quickly through the bloodstream and being absorbed by the cell, delivery of drugs and imaging agents to target sites, minimized drug loss and toxicity, and a long shelf-life. In contrast, its polymeric part responds to external or internal stimuli such as pH, temperature, concentration, enzymes, etc., and allows the drug to be directed to the desired site in a controlled way [123]. Bustamante-Torres et al. developed a highly cross-linked hydrogel system loaded with ciprofloxacin and silver NPs (active biocides). They studied the pH sensitivity of the hydrogel in order to mimic the skin’s physiological pathway for delivering these active compounds. Finally, these hydrogels loaded with these antimicrobial compounds were evaluated against methicillin-resistant *Staphylococcus aureus* and *Escerichia coli*, showing great results. However, it is necessary to perform antimicrobial tests with other pathogens to have a greater spectrum of action. Likewise, the effect it will have on its application on the skin must also be evaluated [125].

**Figure 4:**
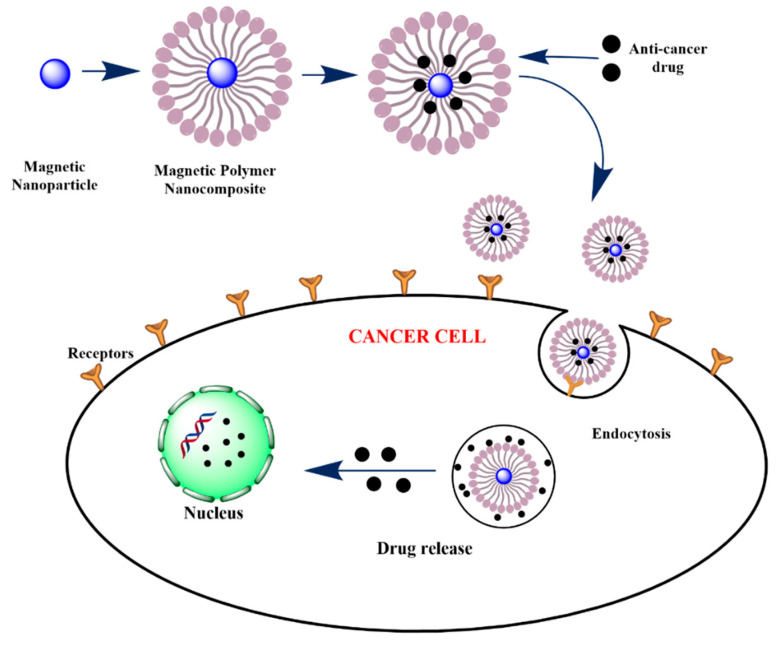
Schematic diagram to describe the synthesis of polymeric MNCs and drug delivery in cancer cells mediated by MNPs.

Polymer nanocomposites are being used for diagnostic and cancer treatment, especially those made of magnetic materials [122,126] (Figure 4). The investigation studied the administration of the anticancer drug 5-fluorouracil using a chitosan/poly (acrylic acid)/Fe_3_O_4_ MNCs hydrogel. The results showed that the stability of the drug dosing for a long time was improved with controlled releases in the conditions of the colon and rectum. The specificity of drug release is what makes this work striking [127]. Additionality, the polymeric MNCs enhanced optical properties such as light absorption and scattering allow the NPs to effectively convert absorbed intense light into localized heat, contributing to thermal phototherapy. Besides, the antineoplastic effect that they possess is used profitably to stop tumor growth [126].

### 6.3. Theranostics

The term “theranostics” defines clinical efforts to develop more specific personalized therapies for various diseases and combine diagnostic and therapeutic capabilities in a single drug [128]. The basic principle stems from the fact that diseases like cancer are highly heterogeneous, and all existing treatments are only effective for a limited subset of patients and at specific stages of the disease. It is hoped that the close integration between diagnosis and treatment can provide patients with a more concrete treatment plan, thus providing a better prognosis [128,129].

Theranostics works like other drugs by interacting with protein molecules called receptors on the cell wall. Different molecules are attracted to different types of receptors. Thus, these types of drugs are linked to molecules that have been selected based on how they interact with the body when there are certain types of cancer. The molecule then transports the drug to the target tumor for diagnosis or treatment. Since healthy cells do not have the same receptors as target cells, the drug will avoid them and will not harm them.

The importance of using MNPs in theranostic field is due to flexibility, sensibility, and effectiveness. Also, MNP can be effectively stimulated from the outside through an appropriate magnetic field [130,131]. Since their size is only tens of nanometers, they can be manipulated to reach various tumors with pore sizes of 100 nm. However, surface area, particle size, and surface charge are some considerations to take into account when manufacturing these types of theranostic, as they determine the pharmacokinetics, toxicity, and biodistribution of the drug [132,133]. Furthermore, studies have shown that the efficiency of administration to a specific site depends on the profile charged MNP, the intensity of the field, the depth of the target tissue, the rate of blood flow, and the vascular supply [132].

Theranostic nanomedicine has the potential to overcome the side effects and the inefficiency of some therapies [134]. They have advanced capabilities targeted delivery, controlled release, higher transport efficiency by endocytosis, smart delivery, synergetic performance, and multimodality diagnosis [135]. For this reason, studies have developed using polymers with magnetic materials like NPs [134,135]. Using MNPs of Fe_3_O_4_ and the carboxymethylcellulose (CMC) biopolymer could induce significant cancer cell death when an alternating magnetic field is applied [136]. For this reason, the CMC-MNP conjugation being promising for the administration of CMC-based targeted drugs, cell imaging, as well as having a high drug loading efficiency and low cellular cytotoxicity.

### 6.4. Imaging

Clinical imaging methods constitute a diagnostic technique for different diseases, including cancer, atherosclerosis, diabetes. However, they do not have enough resolution to have early detection of these [137,138]. Therefore, it is necessary to use a contrast-enhancing agent composed of a signal amplifying material conjugated with a targeting agent to identify molecular markers [138]. MNPs have proven their effectiveness by acting as contrast agents, especially in magnetic resonance imaging (MRI).

For use in biomedicine, MNPs must be tailored to provide both biocompatibility and functionality on their surface. Custom magnetic field gradients are then used to guide them to their destination and provide functionalities either as contrast agents in MRIs or imaging modalities such as magnetic particle imaging. Also, subjecting NPs to a different magnetic field has contributed to this latest technology (magnetic particle imaging) [139].

The use of MNPs in molecular imaging is a relatively new but very active field due to the specific advantages of each material and those derived from its size [140]. For example, due to this nanoscale, the surface-volume ratio is exceptionally high, which translates into the possibility that each NP contains many peptides and antibody carriers; this allows a more significant signal in vivo with less injected probe [141]. In addition, the easy variation of the size of the NPs allows the pharmacokinetics of the probes to be modulated [142]. Besides, studies have shown the use of MNPs made from iron oxide due to their deep tissue imaging capabilities, non-immunogenetic, and low toxicity [143].

Cancer imaging is another application that can benefit from the use of nanomagnetic NPs since these can form clinical images that allow the identification of lesions as small as 2–3 mm [139,144], identify metastases in lymph nodes with a diameter from 5 to 10 mm under magnetic resonance imaging [139,145], in addition to its use it has made it possible to improve the delimitation of the limits of brain tumors and to quantify tumor volumes [139,146]. Furthermore, research suggests that actively targeted MNPs will improve the detection and localization of significant tumors [139].

Achieving adequate image sensitivity for the specific diagnosis of cancer is essential for advancement in medicine, and this can be achieved based on the synergy of MNPs and polymers. For example, research has used poly (L-glutamic acid) (PGA) as the carrier for the polymer polyethylene glycol (PEG) for diagnostic imaging [147]. The resulting MRI images of a tumor show enhanced contrast and a more significant number of observed tumors. On the other hand, the use of poly (styrene-block-allyl alcohol) copolymer together with IONPs allows to obtain triple modal magnetic resonance images and to follow cancer cells in-vivo. This research suggests a method to develop a nanocomposite system with peculiar and interesting integrated functionalities that could be used for biomedical imaging and cancer therapy [148]. Thus, nanocomposites made up of polymers, and MNPs are new camps with some advantages for clinical imaging.

### 6.5. Bioremediation

As mentioned before, nanocomposites have been demonstrated to be efficient in generating products with more efficient reactivity and larger surface area than their bulk phase. Therefore, nanotechnology in bioremediation processes has taken importance in the last years due to its economical, eco-friendly, and self-propelling attributes [149], especially in industrial effluent treatment. For example, magnetite (Fe_3_O_4_) NPs are largely used for biomedical and water treatment applications, among others [59].

In wastewater, there are many contaminants, which can be classified into two main groups: biodegradable organic compounds and non-biodegradable organic compounds. The first can be treated using bacteria, while persistent organic compounds need more advanced treatments than the conventional physicochemical methods of remediation such as adsorption, flocculation, and coagulation, where the use of nanocomposites favors their development [150].

Many studies have proven how MNCs show high adsorption capacities to remove heavy metals, oils, organic solvents, and emerging pollutants from water. It has been found that the use of MNPs enzyme-conjugated has more advantage over metal only [151]; for example, using amino-functionalized MNPs in the laccase has been found out to increase its thermal and operational stability, high volumetric activity, and improve its resistance to extreme conditions [152]. In addition to the potential, they have to decrease environmental contamination from dye industry effluents. Another study has used the enzyme glycerophosphodiesterase immobilized on magnetite NPs to remove toxic compounds like organophosphate pesticides from the environment [153].

The wastewater from petrochemical industries contain many dangerous contaminants such as aliphatic, aromatic, asphaltene and resin hydrocarbons, which have high toxicity, and some of them are mutagenic and carcinogenic [149]. Investigations have found the use of r-Fe_2_O_3_ MNPs in *Comamonas* sp. Células JB is efficient in bioremediation wastewater containing benzene, toluene, ethylbenzene, and xylene (BTEX) compounds. The results obtained from this study suggest that it is a promising technique for improving biocatalysts used in the bioremediation of hazardous wastewater wastes, not just petrochemical wastewater [154]. Also, magnetic shell cross-linked needle-like NPs (MSCKs) removed hydrocarbons present in crude oil successfully with a proportion of 10 mg of oil per 1 mg of MSCK. This study suggests a systematic development of hybrid nanocomposites, which could help the scientific community’s efforts in environmental remediation issues, including oil spill sites following the bulk recovery phase [155].

Another application of MNPs is the chemical oxidation of petroleum hydrocarbons, especially in soil contaminated by waste compounds, where MNP can reduce the toxic effect of the hydrocarbon and support microbial growth. For example, the use of hydrogen peroxide (H_2_O_2_) and iron oxide (FeO) in a 33.7:1 ratio removed up to 91% of the total petroleum hydrocarbons within four hours [149]. Besides, the use of magnetite NPs and H_2_O_2_ in the molar ratio of 17.5:1, leading to the removal of 74.20% TPH acting as a catalyst in the oxidation process to improve hydroxyl radical production [156]. On the other hand, cobalt and manganese NPs have successfully reduced polycyclic aromatic and heteroaromatic hydrocarbons [157].

The combination of technology that uses nanocomposites can provide better characteristics and optimization of existing processes. In the case of bioremediation, the use of an electrochemical biosensor based on the immobilization of laccase on magnetic core-shell (Fe_3_O_4_–SiO_2_) NPs were combined with artificial neural networks for the determination of catechol concentration in it. The study results showed that combining an amperometric enzyme sensor and an artificial neural networks was effective because it presents sensitivity and is a robust method in the quantitative study of the composting system [158]. These and many more applications in bioremediation can be achieved with MNPs, and there is a whole world of possibilities awaiting.

## 7. Conclusions

The interaction between polymers and reinforcing nanofillers is a promising research topic for many applications. Polymers show attractive versatility to form PMs due to their surface, thermal response, and polymerization mechanisms. Some of the most famous PMs are hydrogels due to their high capacity to absorb solvents, including water and drugs. Typically, polymers are reinforced by fillers such as NPs forming composites arising from the diffusion with the matrix, mechanical interlocking, or intermolecular bonding. The magnetic approach is led by MNPs, which confer magnetic properties such as ferromagnetism, ferrimagnetism, and superparamagnetic behavior to the PNCs, in the presence or absence of a magnetic field. A significant effort has focused on MNPs coated by polymers because they protect and prevent the oxidation of the NPs. The interaction of MNPs and polymer improves their biocompatibility and creates a multifunctional system by adding reactive and bioactive groups on the surface of the composite material, thereby expanding the scope of application. Commonly, MNCs present superparamagnetic behavior that can be used for controlled and targeted drug delivery, through the use of an external magnetic field and thus treat difficult diseases, allowing them to be useful in biomedicine.

Moreover, polymer nanocomposites have been reported to enhance the rigidity, mechanical strength, high-temperature resistance, weight reduction, corrosion resistance, and electrical conductivity of matrix materials. The treatment of fatal diseases and their application in environmental restoration are areas that are expected to be further developed in the next few years. There is no doubt that betting on these relative innovations will be a practical challenge. Although there are gaps to cross, the advances are increasingly accelerated, opening up space for development.

## Data Availability

Not applicable.
